# Glaucoma Medication Preferences among Glaucoma Specialists in Mexico

**DOI:** 10.5005/jp-journals-10028-1232

**Published:** 2017-10-27

**Authors:** Gabriel Lazcano-Gomez, Daniela Alvarez-Ascencio, Cindy Haro-Zuno, Mauricio Turati-Acosta, Magdalena Garcia-Huerta, Jesus Jimenez-Arroyo, Rafael Castañeda-Diez, Armando Castillejos-Chevez, Roberto Gonzalez-Salinas, Francisca Dominguez-Dueñas, Jesus Jimenez-Roman

**Affiliations:** 1 Associate Professor, Department of Glaucoma, Association to Avoid Blindness in Mexico, IAP, Mexico City, Mexico; 2 Fellow, Department of Glaucoma, Association to Avoid Blindness in Mexico, IAP, Mexico City, Mexico; 3 Fellow, Department of Glaucoma, Association to Avoid Blindness in Mexico, IAP, Mexico City, Mexico; 4 Professor, Department of Glaucoma, Association to Avoid Blindness in Mexico, IAP, Mexico City, Mexico; 5 Associate Professor, Department of Glaucoma, Association to Avoid Blindness in Mexico, IAP, Mexico City, Mexico; 6 Associate Professor, Department of Glaucoma, Association to Avoid Blindness in Mexico, IAP, Mexico City, Mexico; 7 Associate Professor, Department of Glaucoma, Association to Avoid Blindness in Mexico, IAP, Mexico City, Mexico; 8 Associate Professor, Department of Glaucoma, Association to Avoid Blindness in Mexico, IAP, Mexico City, Mexico; 9 Associate Researcher, Department of Biomedical Research, University of Queretaro Queretaro, Mexico; 10 Head, Department of Glaucoma, National Institute of Rehabilitation Mexico City, Mexico; 11 Head, Department of Glaucoma, Association to Avoid Blindness in Mexico, IAP, Mexico City, Mexico

**Keywords:** Brands, Generic, Glaucoma medications, Preferences, Survey.

## Abstract

**Aim:**

To determine the glaucoma specialists’ preferences for the different brands of topical glaucoma medications available in Mexico.

**Materials and methods:**

A web-based survey was sent to 150 board-certified glaucoma specialists in Mexico, with 14 questions related to brand preferences for all glaucoma medications available in Mexico. Participants were asked to select each glaucoma medication class by brand and to state the factors leading to their choice.

**Results:**

Data from 111 (74%) glaucoma specialists were collected. Imot (timolol 0.5%; Sophia, Mexico) was the preferred brand for the beta-blockers (BB) class by 71% (n = 79) of the participants. Azopt (brinzolamide 1%; Alcon Lab, US) was the preferred carbonic anhydrase inhibitor (CAI) by 54% (n = 60) of the glaucoma specialists. Lumigan (bimatoprost 0.01% and 0.03%; Allergan Inc., U.S.) was the first choice for the prostaglandin analogues (PGAs) in 62% (n = 70) of the answers. The most frequently prescribed alpha-agonist (AA) was Agglad (brimonidine 0.2%; Sophia Lab, Mexico) in 44% (n = 49) of the answers. Medication accessibility (31%), cost (29%), and recommended dose (23%) were the three main factors influencing the glaucoma specialists’ preferences.

**Conclusion:**

Medication cost and accessibility, as well as posology, remain the main factors influencing brand preferences among glaucoma doctors. In our professional opinion, the therapeutic effect must be the leading factor when prescribing topical medications in the daily practice, so that patients receive the best treatment option.

**Clinical significance:**

This survey provides an understanding of the decision-making process when prescribing glaucoma medications by glaucoma specialists in a Latin American developing country. Ideally, patient treatment should be individualized and aimed to achieve the best results possible for their specific condition.

**How to cite this article:** Lazcano-Gomez G, Alvarez-Ascencio D, Haro-Zuno C, Turati-Acosta M, Garcia-Huerta M, Jimenez-Arroyo J, Castañeda-Diez R, Castillejos-Chevez A, Gonzalez-Salinas R, Dominguez-Dueñas F, Jimenez-Roman J. Glaucoma Medication Preferences among Glaucoma Specialists in Mexico. J Curr Glaucoma Pract 2017;11(3):97-100.

## INTRODUCTION

Glaucoma is the leading cause of irreversible blindness worldwide.^1^ It is a chronic and progressive disease, which to this day has only one modifiable risk factor, lowering the intraocular pressure (IOP). This has been shown to slow or halt the progression of the optic nerve (ON) degeneration.^2^ Even though a number of treatment options (lasers and novel surgical techniques) have been developed, topical medications remain the first-line therapy for glaucoma in the majority of cases.^3,4^

Currently, the pharmaceutical market provides physicians with a broad spectrum of brands for each class of glaucoma medications. Brands can differ not only in price, but also in posology, preservative, and bottle design, which may affect treatment outcomes. Even though the therapeutic effect should be the main reason when prescribing specific brands,^5,6^ physicians’ preferences for certain brands are also determined by the drug availability, cost, patient’s preference, and insurance coverage, and in some cases are influenced by the exposure to the pharmaceutical companies.^7^

Many regulations have been developed to prevent the risks and avoid doctors’ exposure to certain pharmaceutical company influences. These regulations have benefited both doctors and patients, in reducing the number of malpractice lawsuits and misconducts by the medical and pharmaceutical community.^8^

There has not been a descriptive review of brand preferences among glaucoma specialists in Latin America, which has a population with high incidence of open-angle glaucoma (OAG).^9^ This study aims to determine the preferences of glaucoma specialists for the different brands of all topical glaucoma medications available in Mexico, and it was conducted from May to June 2016.

## MATERIALS AND METHODS

This cross-sectional study was approved by the Internal Review Board of the “Asociacion Para Evitar la Ceguera en Mexico” Hospital. We created a web-based questionnaire (SurveyMonkey®), with 14 questions related to brand preferences for all the glaucoma medications available in Mexico. The survey was delivered to 150 board-certified glaucoma specialists in Mexico using the “Mexican Ophthalmology Society” database and with the authorization of the “Mexican College of Glaucoma.” The survey did not include any personal information to maintain the confidentiality of the study participants.

The survey included all the glaucoma medications available on the Mexican pharmaceutical market, including generic medications. All available brands were categorized by (1) glaucoma medication class: BB, CAIs, PGAs, and AAs; and (2) fixed combination drug type: Double fixed combination and triple fixed combination. The participants were asked to choose their preferred brand for each medication class and type. Assessment of the factors influencing their preferences, such as cost, availability, bottle design, insurance, recommended dose, personal preference, and relationship with the pharmaceutical company, was also included.

Data were collected using the same online program and statistical analyses were performed using SAS software version 9.4 (SAS Institute Inc, Cary, NC).

**Graph 1:**
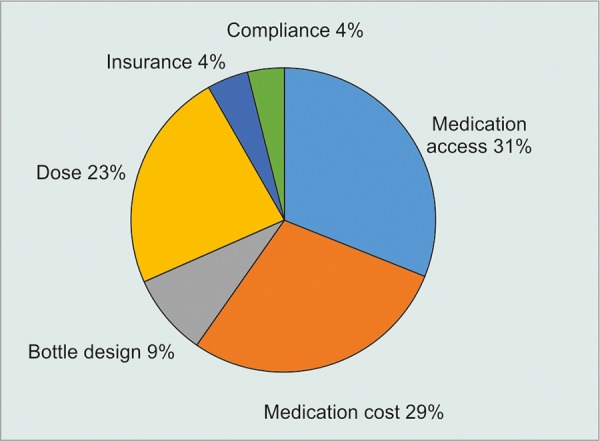
Factors leading to medication choice

## RESULTS

**Graph 2:**
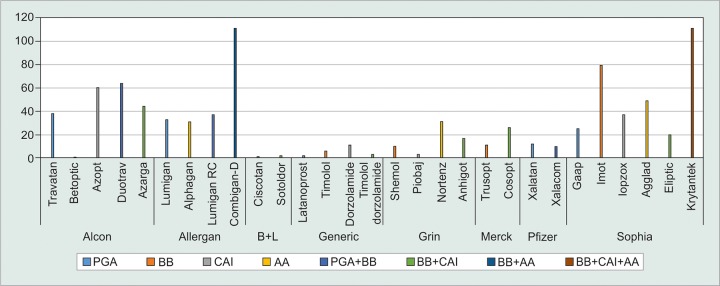
Glaucoma medication preferences by type and brand

Data from 111 (74%) glaucoma specialists were collected. Imot (timolol 0.5%; Sophia Laboratories, Guadalajara, Mexico) was the preferred brand for BB by 71% (n = 79) of the participants. Azopt (brinzolamide 1%; Alcon Lab, U.S.) was the preferred CAI by 54% (n = 60) of the cases. Lumigan (bimatoprost 0.01% and 0.03%; Allergan Inc., U.S.) was the first choice for the PGAs in 62% (n = 70) of the answers. The most frequently prescribed AA was Agglad (brimonidine 0.2%; Sophia Lab, Mexico) in 44% (n = 49) of the answers. Combigan-D (timolol 0.2%/ brimonidine 0.5%; Allergan Inc., U.S.) and Krytantek (timolol 0.5%/dorzolamide 2%/brimonidine 0.2%; Sophia Lab, Mexico) were the preferred fixed combinations by 100% (n = 111) of the physicians. Commercial brands were preferred over generics for all glaucoma medications. The preferences for each brand are shown in Graph 1. Medication access (31%, n = 64), cost (29%, n = 59), and recommended dose (23%, n = 48) were the three main factors influencing the preferences. The factors that lead to the choice of each brand are shown in Graph 2.

## DISCUSSION

As previously described, Alcon Laboratories (Fort Worth, Texas, US) and Sophia Laboratories (Guadalajara, Mexico) lead the glaucoma drugs market in Mexico. Lumigan (bimatoprost 0.01% and 0.03%; Allergan Inc., U.S.), Agglad (brimonidine 0.2%; Sophia Lab, Mexico), and Duotrav (travoprost 0.004%/timolol 0.5%; Alcon Lab, US) were selected as the first option when prescribing PGA, AA, and a double fixed combination of PGA+BB respectively, in this study. Imot (timolol 0.5%; Sophia Lab, Mexico), Azarga (brinzolamide 1%/timolol 0.5%; Alcon Lab, US), and Krytantek (timolol 0.5%/dorzolamide 2%/ brimonidine 0.2%; Sophia Lab, Mexico) were selected as the first option when prescribing BB, BB+CAI, and a triple fixed combination of BB+CAI+AA. Krytantek is the only triple fixed combination available in the Mexican pharmaceutical market.

Access to the medications remains an important issue when prescribing them in the developing countries. The availability of certain drugs may not be widespread through all the regions and could lead to difficulties with treatment compliance.^10^ Medication cost is also one of the leading factors, influencing physicians’ preferences, especially in countries like Mexico, where most patients do not have health insurance.^11^ Even though 30% of the physicians considered the cost to be the most important factor when prescribing a medication, the preferred brands in this study were the most expensive options available, as reported in our previous study on the glaucoma therapy cost in Mexico.^5^

Pharmaceutical companies use marketing strategies in order to influence physicians’ choices when prescribing medications;^12^ the more expensive the strategies are the more effective they become.^13^ Other influencing factors include good rapport with the pharmaceutical company representatives, company’s reputation, promotional materials, gifts, and other incentives.^14,15^

Product quality and therapeutic effect still need to be the main reason to prescribe a medication of any kind, regardless of the strategies used to promote drugs.^16^

Generic drugs were preferred as the first therapeutic option for glaucoma patients in only 19% of the participants (n = 22), showing that general skepticism related to generic medications is still common. Prescribing generic drugs continues to be an underused strategy that has the potential to increase value of care, reduce health care costs, and improve medication adherence and, ultimately, clinical results especially in developing countries.^17^ Unfortunately, unlike their brand-name counterparts, generic drugs are not marketed, leaving physicians to learn about them from colleagues, literature, pharmaceutical representatives, and/or patients themselves.^18,19^

Collecting research data from a web-based survey has certain limitations when analyzing the results. This survey was directed only to board-certified glaucoma specialists, but since noncertified glaucoma specialists and general ophthalmologists can also prescribe glaucoma medications, the overall preferences of the Mexican ophthalmologists for glaucoma medications available in Mexico may not be reflected in the results of this study.

## CONCLUSION

Many factors play a role in the prescription preferences and patterns by glaucoma specialists. Even though pharmaceutical marketing can have positive effects regarding medical prescriptions, physicians should be aware of direct and indirect influences on their practice and therapeutic decisions. Medication cost and accessibility, as well as posology, remain the main factors influencing brand preferences among doctors. Based on our professional opinion, the therapeutic effect must be the leading factor when prescribing medications in the daily practice, so that patients receive the best treatment modality for their diseases. The results reported in this study demonstrate that although in a small percentage, many factors, such as pharmaceutical marketing strategies and compliance with pharmaceuticals, continue to play an important role when deciding which brand should be prescribed for glaucoma patients.

## CLINICAL SIGNIFICANCE

This survey provides an understanding on the decision-making process when prescribing glaucoma medications by specialists in a Latin American developing country. Ideally, patient treatment should be individualized and aimed to achieve the best results possible for their specific condition.
